# Research on the RZB-Type Three-Dimensional Drilling Strain Measurement System

**DOI:** 10.3390/s24123954

**Published:** 2024-06-18

**Authors:** Zheng Chen, Hong Li, Yunkai Dong, Wenbo Wang, Liheng Wu, Weiwei Zhan

**Affiliations:** National Institute of Natural Hazards, Beijing 100085, China

**Keywords:** borehole strain meter, three-dimensional borehole strain measurement, vertical strain measurement

## Abstract

Borehole strain gauges play a crucial role in geophysical, seismological, and crustal dynamics studies. While existing borehole strain gauges are proficient in measuring horizontal strains within vertical boreholes, their effectiveness in capturing vertical and oblique strains is limited due to technical constraints arising from the cylindrical probe’s characteristics. However, the accurate measurement of three-dimensional strain is essential for a comprehensive understanding of crustal tectonics, dynamics, and geophysics, particularly considering the diverse geological structures and force sources within the crustal medium. In this study, we present a novel approach to address this challenge by enhancing an existing horizontal-component borehole strain gauge with a bellows structure and line strain measurement technology to enable vertical and borehole oblique strain measurements. Integrating these enhancements with horizontal strain measurement capabilities enables comprehensive three-dimensional borehole strain measurements within the same hole section. The system was deployed and tested at the Gongxian seismic station in Sichuan Province. Clear observations of solid tides were recorded across horizontal, oblique, and vertical measurement units, with the tidal morphology and amplitude being consistent with the theoretical calculations. The achieved measurement sensitivity of 10-10 meets the requirements for borehole strain measurement, enabling the characterization of three-dimensional strain states within boreholes through association methods.

## 1. Introduction

A borehole strain gauge is a high-precision instrument for measuring additional strain in the crust, and it is an important means of studying geophysics, seismology, and crustal dynamics. At present, research teams from various countries have developed various models of borehole strain gauges. China’s National Geophysical Network uses various models of borehole strain gauges, such as RZB-2, YRY-4, and SKZ-3; the PBO program in the United States uses the BTSM-type borehole tensor strain gauge.

**Figure 1:**
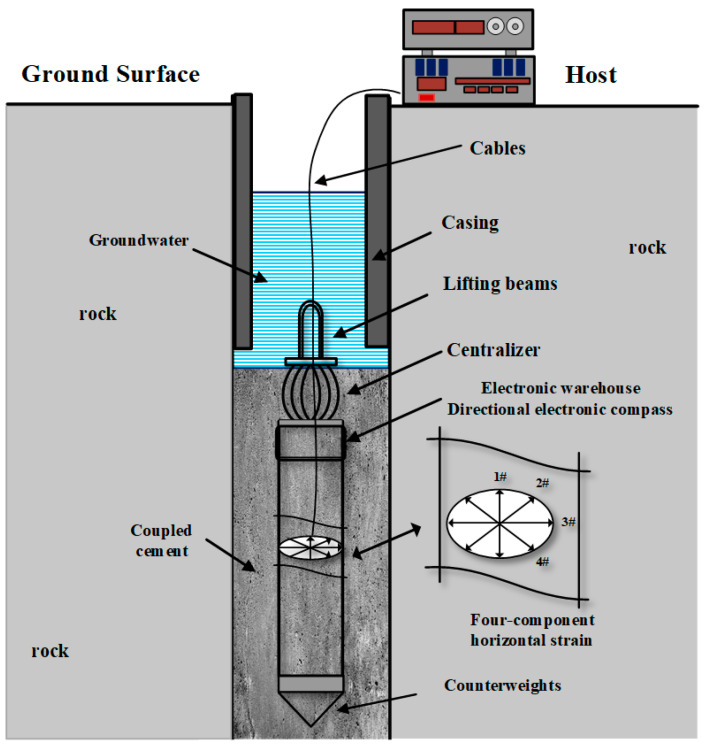
Schematic diagram of borehole strain observation system.

These types of borehole strain gauges are all horizontal component strain gauges that can observe crustal strain in four horizontal directions. Drilling strain gauges generally consist of strain probes, hosts, and data collectors. A strain probe is a sensing unit for strain measurement that is installed in a borehole. The host is a measurement and control circuit unit; the data collector is a unit for data collection, storage, and transmission. Most strain probes adopt the mechanical structure shown in Figure 1 utilizing the isotropic mechanical properties of the horizontal section of cylindrical steel cylinders. Displacement sensors are uniformly arranged at a 45° angle on the horizontal section inside the steel cylinder, and both ends of the sensors are fixed on the inner wall of the steel cylinder. When the steel cylinder undergoes compression or tensile deformation, the sensors can accurately measure the displacement deformation of the horizontal section of the steel cylinder. In actual observation, strain probes are installed in boreholes and coupled with special cement. The probe achieves welding coupling boundary conditions by coupling cement with the rock layers inside the borehole. The deformation of the borehole bedrock is transmitted to the steel cylinder through the coupling cement. The displacement sensor can accurately measure the displacement in each direction and then remove it from the measured position to measure the baseline, obtaining the strain value [1,2,3,4,5,6,7,8].

**Figure 2:**
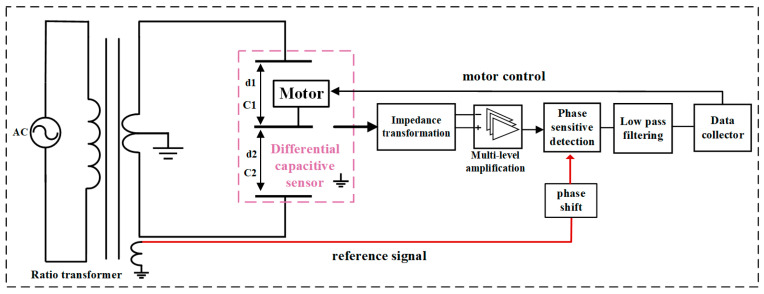
Schematic diagram of differential capacitance micro-displacement sensing circuit.

To obtain highly sensitive strain measurement, drilling strain gauges generally use a differential capacitance micro-displacement sensor with a three-pole plate, and the measurement circuit is shown in Figure 2. The differential capacitance sensor is composed of three parallel metal plates with fixed spacing between the two plates. The middle plate can move with the outer cylinder of the probe. The differential capacitor and the transformer with the center tap form a measurement bridge. The unbalanced bridge signal output by the pole plate in the differential capacitor is amplified, phase-sensitive detected, and low-pass filtered before being output to the data collector. The middle plate of the sensor will be connected to the mechanical adjustment mechanism. When the displacement of the middle plate is too large and the unbalanced signal of the bridge circuit is limited, the adjustment motor can be used to pull the middle plate back to the center position.

At present, borehole strain gauges for horizontal observation are relatively mature and widely used in geophysical networks. However, with the deepening of geophysical research, research on the destruction of the North China Craton in China has shown that the deep crust undergoes non-horizontal deformation such as distortion and folding under tensile compression [9,10]. The GPS surface deformation data of the Longmenshan Fault Zone reveals that the Wenchuan 8.0 earthquake caused a large-scale, far-field co- seismic displacement field in the fault zone, with maximum vertical and horizontal offsets reaching 6 m and 4.9 m, respectively. The investigation of surface coseismic displacement at the Wenchuan earthquake site also found that the maximum vertical displacement in the Zoujiayuan area of Shaba Village is 9 ± 0.5 m. These studies indicate that, due to the complexity of geological structures and the diversity of force sources, tectonic activity and crustal deformation are not limited to horizontal movement [11,12,13,14,15]. To conduct in-depth and comprehensive research on crustal tectonic movement and deformation, borehole strain observation cannot be limited to the horizontal direction and needs to be extended to three-dimensional strain observation. In this paper, based on the RZB-type component (horizontal) borehole strain meter, we designed bellows structures for borehole vertical strain measurement units and oblique line strain measurement units. Integrated with the horizontal strain measurement probe, these components constitute the RZB three-dimensional borehole strain observing system. Field observations at the Gongxian seismic station in Sichuan Province demonstrate that the horizontal, vertical, and oblique strain measurement units of the system can accurately record solid tidal data. The observed tidal morphology and amplitude align with theoretical predictions, meeting the requirements of geophysical field observations and addressing three-dimensional strain problems.

## 2. Structure of RZB Three-Dimensional Strain Probe

**Figure 3:**
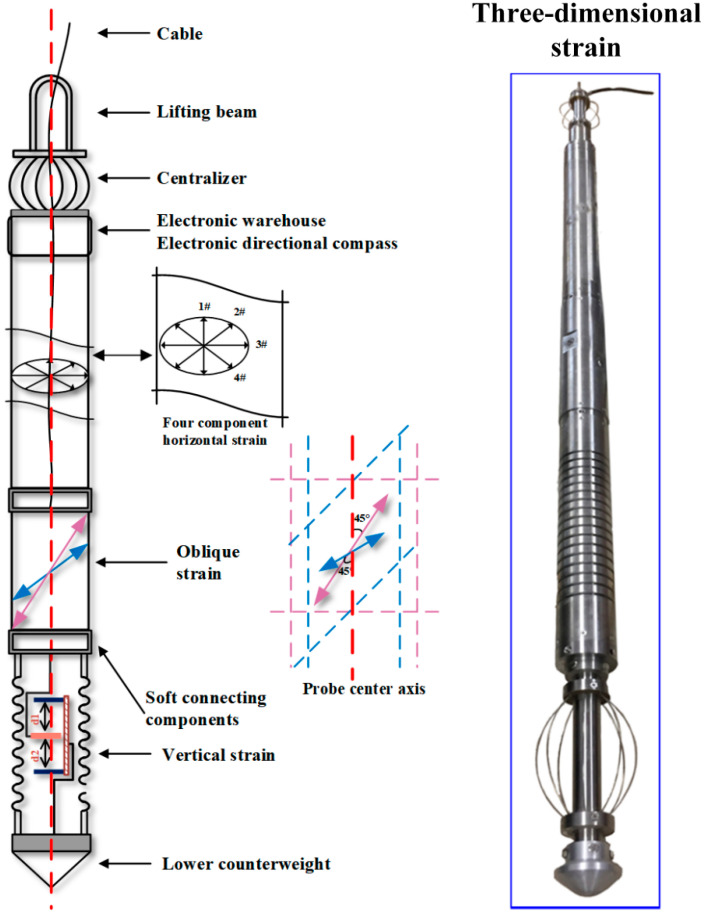
RZB deep wideband deformation integrated observing system.

The strain probe of the RZB three-dimensional drilling strain observation system incorporates vertical and oblique strain measurement units based on the horizontal strain probe. As shown in Figure 3, the oblique and vertical strain probes are positioned at the lower end of the horizontal strain probe. Soft components are used to connect the vertical strain probe and the oblique strain probe, ensuring that they are unaffected by the deformation of the strain probe above the measurement baseline. At the upper end of the horizontal strain probe, the electronic compartment and directional compass are situated. The uppermost section of the probe includes the armature, electronic compartment, and downhole lifting beam [16]. These probes are installed in boreholes ranging from 0 to 400 m deep in selected intact bedrock layers and cast with coupling cement. Welded coupling to the borehole wall ensures boundary conditions. The modulus of elasticity of the borehole bedrock generally exceeds 10^4^ MPa, while that of the coupling cement is slightly lower. However, the equivalent modulus of each strain probe component remains within 10^3^ MPa, enabling the coupling cement to transfer bedrock deformation to the strain probe with minimal loss. The coupling cement, with its expansion function, not only enhances coupling effectiveness but also applies pre-stress to the probe during coupling. This ensures that the probe is pressurized and deformed during coupling, facilitating the measurement of tensile bedrock deformation.

### 2.1. Horizontal Strain Probe Structure

**Figure 4:**
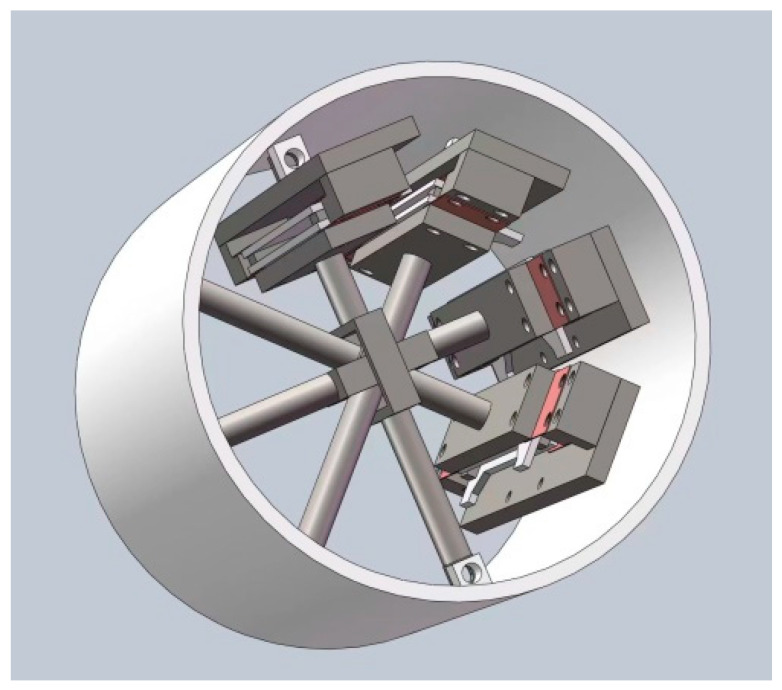
The structure of a horizontal strain probe.

Horizontal strain probes utilize the principle of horizontal isotropy of a vertical, infinite-length steel cylinder to install four-component micro-displacement sensors in the middle of the cylinder at an angle of 45° to the same plane in sequence. The strain probe uses a steel cylinder with an outer diameter of 100 mm and a length of 1 m. The length of the cylinder is 10 times the diameter, allowing for the elimination of end effects as much as possible. As shown in Figure 4, both ends of the sensor are fixed to the inner wall of the steel cylinder through a drive rod, enabling the sensor to accurately measure the horizontal displacement of the probe when the probe deforms with the drilled hole. After measuring the horizontal strain of the steel cylinder in the same plane, the drive rods are bound to cross in the center part of the horizontal cross section, making the drive rods symmetrical hollow structures at the crossing point, and they can be interspersed with each other to go around [17].

### 2.2. The Structure of a Vertical Strain Probe

In borehole strain measurement, the strain probe is the sensing unit, coupling cement is a deformation transmission medium, and the rock mass is the elastic body to be measured. According to the principle of deformation measurement, the sensing unit and coupling cement need to be softer than the rock mass for the deformation of the rock mass to be transmitted to the sensing unit without loss. For vertical strain measurement in a borehole, the axial equivalent elastic modulus of the vertical strain probe is required to be one order of magnitude smaller than that of the coupling cement and rock, and the elastic modulus in the horizontal direction should be higher than that in the vertical direction, comparable to that of the coupling cement. The deformation in the horizontal direction has less influence on the vertical measurement. As the strain probe is installed in the vertical shaft, the cylindrical structure of the steel cylinder makes its equivalent modulus of elasticity in the vertical direction much higher than that of the coupling cement and the surrounding bedrock, while the equivalent modulus of elasticity in the horizontal direction is lower. The horizontal deformation of the borehole has a greater effect on the vertical strain measurement, so it is not conducive to the measurement of vertical strain.

The outer cylinder of the RZB-type vertical strain measurement unit adopts a bellows structure, and the sensor is installed vertically in the probe, with both ends of the sensor fixed by a transmission rod. The two ends of the sensor are fixed to the end of the probe through the drive rod, enabling the accurate measurement of the vertical deformation of the probe [18,19]. As shown in Figure 3, the elastic modulus of the bellows in the vertical and horizontal directions is determined by parameters such as the wave thickness of the corrugation, wave pitch, inner and outer diameters of the probe, and material. The corrugated structure can effectively reduce the axial equivalent elastic modulus of the probe while increasing the thickness of the probe, and adjusting the wave spacing and wave thickness can strengthen the horizontal equivalent elastic modulus. The bellows can be regarded as a column spring, which is easy to deform in the axial direction while being stiffer and less prone to deformation in the horizontal direction.

The vertical equivalent modulus of elasticity of the RZB-type vertical strain measurement unit is about 10^3^ MPa, one order of magnitude lower than that of the coupling cement, while the horizontal equivalent modulus of elasticity exceeds 10^4^ MPa, one order of magnitude higher than that of the vertical direction. The deformation in the horizontal direction has a smaller effect on the vertical deformation, aligning with the requirements of vertical line strain measurement.

### 2.3. Inclined Strain Probe Structural Units

**Figure 5:**
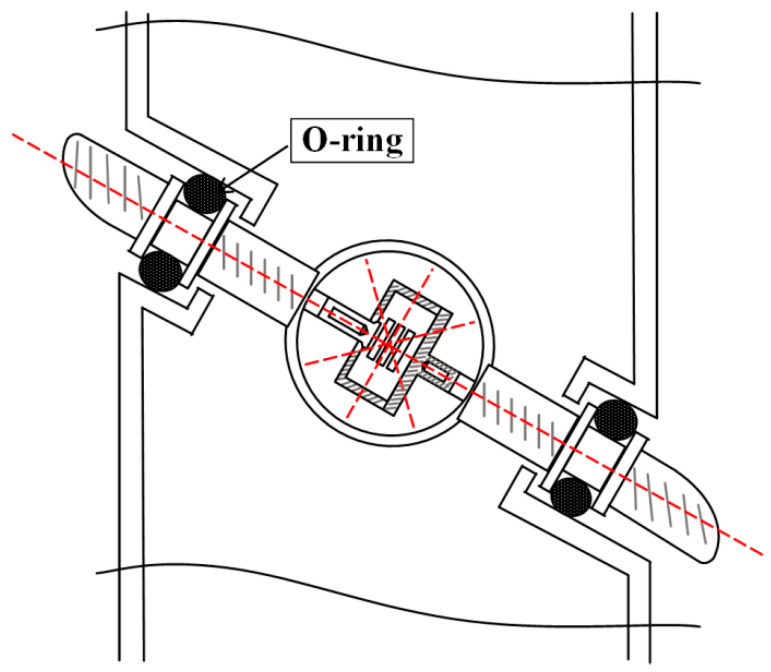
The structure of a diagonal strain probe.

The oblique strain probe adopts a line strain measurement structure similar to that of a telescope. The diagonal strain sensor is installed in a metal ring, and the equivalent modulus of the ring can be precisely controlled according to the thickness and diameter of the ring, ensuring that the strain sensor achieves a hardness close to that of the coupling cement. Guide rods extend obliquely from both sides of the ring, sealing with Viton “O” rings where they meet the outer barrel of the probe. These rubber sealing rings serve both as seals and fixed parts of the sensor, securing it inside the probe by compressing against the guide rail. During the coupling process, as the cement binds to the probe, the head of the guide rail and the cement are completely welded together. While the cement deforms in the direction of the guide rod to the sensor, the soft connection between the guide rod and the probe barrel through the rubber ring allows for overall rotation and deflection without affecting radial displacement. This ensures that the sensor can elongate or shorten as required for the measurement of line strain. (See Figure 5).

### 2.4. Theoretical Model of Three-Dimensional Borehole Strain Observation

**Figure 6:**
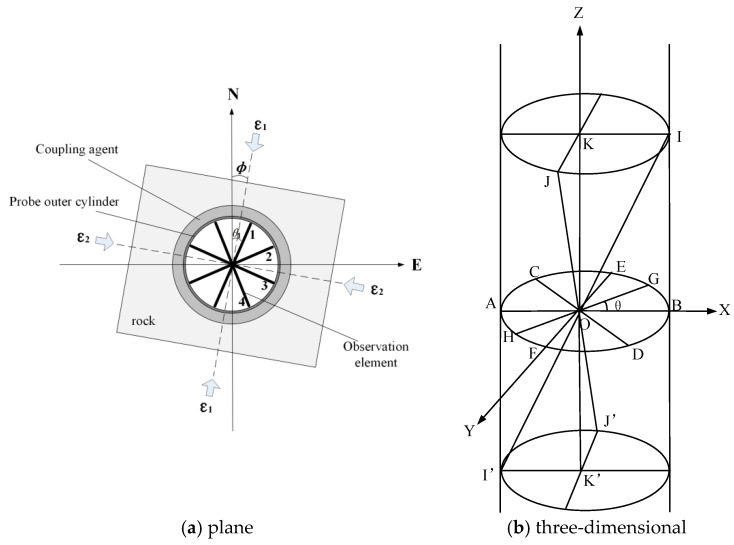
The three-dimensional strain mechanics model of the borehole.

Two-dimensional (horizontal) borehole strain observation generally adopts the plane stress theory in elastic mechanics, as shown in Figure 6a. The strain value of the observation element of the borehole strain gauge is represented by Equation (1) below [20,21,22]:

(1) $$S_{i}=A\left(\varepsilon_{1}+\varepsilon_{2}\right)+B\left(\varepsilon_{1}-\varepsilon_{2}\right)\cos2\left(\theta_{i}-\varphi \right)$$

where $S_{i}$ is the strain value of the four observation elements of the borehole strain gauge (i = 1,2,3,4), $\theta_{i}$ is the angle of the observation elements counterclockwise from the north direction, $\varepsilon_{1}$ and $\varepsilon_{2}$ are the horizontal maximum principal strain and the horizontal minimum principal strain, and *φ* is the angle of the horizontal maximum principal strain counterclockwise from the north direction.

*A* and *B* are the coupling coefficients. In the case of an empty borehole, without considering the influence of the probe outer barrel and the coupler, *A* and *B* are only related to the Poisson’s ratio of the surrounding rock. In the case of a double-nested case, considering the influence of the probe outer barrel and the coupler, *A* and *B* have a relationship with the modulus of elasticity and Poisson’s ratio of the surrounding rock, the coupling layer, and the probe outer barrel.

The three-dimensional borehole strain observation considers three stress components related to the vertical direction in addition to the three stress components in the horizontal direction. Let the three-dimensional stress state of the rock be ($\sigma_{x},\;\sigma_{x},\;\sigma_{x},\;\tau_{xy},\;\tau_{yz},\;\tau_{zx}$), as shown in Figure 6b. There is a right-angled coordinate system *OXYZ* and a borehole with a radius *R*. The origin of the coordinate system *O* is located in the central axis of the borehole, and the *Z*-axis is in the axial direction along the borehole. The surrounding rock is assumed to be an ideal elastic body. According to the theory of borehole deformation used in the field of earth stress measurement, the displacement of any point in the borehole is given by the following [23]:

(2a) $$u=\frac{R}{E}\left[\left(\sigma_{x}+\sigma_{y}\right)+2\left(1-\mu^{2}\right)\left(\sigma_{x}-\sigma_{y}\right)cos2\theta +4\left(1-\mu^{2}\right)\tau_{xy}sin2\theta -\mu \sigma_{z}\right]$$

(2b) $$v=-\frac{R}{E}(1+\mu )(2-\mu )\left[\left(\sigma_{x}-\sigma_{y}\right)sin2\theta -2\tau_{xy}cos2\theta \right]$$

(2c) $$w=4\frac{R}{E}\left(1+\mu \right)\left(\tau_{yz}\sin\theta +\tau_{zx}\cos\theta \right)+\frac{z}{E}\left[\sigma_{z}-\mu \left(\sigma_{x}+\sigma_{y}\right)\right]$$

where u, v, and w are the radial, axial, and vertical displacements on the borehole wall, respectively. E and μ are the modulus of elasticity and Poisson’s ratio of the rock, respectively, and θ is the counterclockwise angle from the *X*-axis to the direction of the measurement point in the horizontal plane.

For the RZB-type three-dimensional strain probe, *AB*, *CD*, *EF*, and *GH* in Figure 6b represent the four-component horizontal strain observation units, and the horizontal observation baseline is *2R. KK*’ represents the vertical strain observation unit, and the vertical observation baseline is *2W*. *II*’ and *JJ*’ represent the two oblique strain observation units, and the length of the oblique observation baseline is *2L*. According to Formula (2a) for radial displacement u at any point on the borehole wall, the strain value of the horizontal observation unit can be obtained as follows:

(3) $$S_{u}=\frac{u}{R}=\frac{1}{E}\left[\left(\sigma_{x}+\sigma_{y}\right)+2\left(1-\mu^{2}\right)\left(\sigma_{x}-\sigma_{y}\right)cos2\theta +4\left(1-\mu^{2}\right)\tau_{xy}sin2\theta -\mu \sigma_{z}\right]$$

The strain value of the vertical observation unit is

(4) $$S_{w}=\frac{w}{W}=\frac{1}{E}\left[\sigma_{z}-\mu \left(\sigma_{x}+\sigma_{y}\right)\right]+\frac{4R}{EW}\left(1+\mu \right)\tau_{yz}\sin\theta +\frac{4R}{EW}\left(1+\mu \right)\tau_{zx}\cos\theta$$

Since the vertical observation unit is insensitive to $\tau_{yz}$ and $\tau_{zx}$, the strain value of the vertical observation unit can be simplified as

(5) $$S_{w}=\frac{1}{E}\left[\sigma_{z}-\mu \left(\sigma_{x}+\sigma_{y}\right)\right]$$

For the oblique observation unit, according to the theory of ground stress measurement by the borehole wall displacement method, the displacement between two points within the borehole wall that are not in the same horizontal plane can be derived from the following equation [24]:

(6) $$∆L=\sqrt{{\left(R+u\right)}^{2}+v^{2}+{\left(W+w\right)}^{2}}-\sqrt{R^{2}+W^{2}}\approx \frac{Ru+Ww}{L}$$

Substituting u, v, and w into the above equation yields the strain value of the oblique observation unit as follows:

(7) $$\begin{array}{l} S_{l}=\frac{∆L}{L}=\frac{R^{2}}{EL^{2}}[\left(1-\mu \frac{W^{2}}{R^{2}}\right)\left(\sigma_{x}+\sigma_{y}\right)+\left(\frac{W^{2}}{R^{2}}-\mu \right)\sigma_{z}+2\left(1-\mu^{2}\right)\left(\sigma_{x}-\sigma_{y}\right)cos2\theta +4\left(1-\mu^{2}\right)\tau_{xy}sin2\theta \\ \;\;\;\;\;\;\;\;\;\;\;\;+4\frac{W}{R}\left(1+\mu \right)\tau_{yz}\sin\theta +4\frac{W}{R}\left(1+\mu \right)\tau_{zx}\cos\theta ] \end{array}$$

The RZB-type three-dimensional strain probe has seven observed components, denoted as $S_{i}(wherei=1,2,\ldots 7)$. According to the above equations, the values of the final observed seven channels have the following relationship:

$$S_{i}=m_{1}\left(\sigma_{x}+\sigma_{y}\right)+m_{2}\left(\sigma_{x}-\sigma_{y}\right)cos2\theta_{i}+m_{3}\sigma_{z}+m_{4}\tau_{xy}sin2\theta_{i}\;(i=1,\;2,\;3,\;4)$$

(8) $$S_{i}=m_{5}\left(\sigma_{x}+\sigma_{y}\right)+m_{6}\sigma_{z}\;(i=5)$$

$$S_{i}=m_{7}\left(\sigma_{x}+\sigma_{y}\right)+m_{8}\left(\sigma_{x}-\sigma_{y}\right)cos2\theta_{i}+m_{9}\sigma_{z}+m_{10}\tau_{xy}sin2\theta_{i}+m_{11}\tau_{yz}\sin\theta_{i}+m_{12}\tau_{zx}\cos\theta_{i}\;(i=6,7)$$

The three-dimensional ground stress state can be solved based on the above system of eight equations, where the coefficients $m_{j}(j=1,2,\ldots 12)$ are related to the modulus of elasticity of the surrounding rock E, Poisson’s ratio μ, as well as the length of each observation baseline.

The three-dimensional ground stress state can be solved based on the above system of eight equations, where the coefficients $m_{j}(j=1,2,\ldots 12)$ are related to the modulus of elasticity of the surrounding rock E, Poisson’s ratio μ, as well as the length of each observation baseline.

## 3. Differential Capacitance Microdisplacement Measurement Systems

Horizontal, vertical, and oblique strain probes all use capacitive microdisplacement transducers with ratiometric bridges for high-sensitivity strain measurements (10^−10^), enabling the recording of clear solid tides, strained seismic waves, a co-seismic response, and crustal creep.

### 3.1. Differential Capacitance Displacement Sensor and Ratiometric Bridge Measurement Circuitry

**Figure 7:**
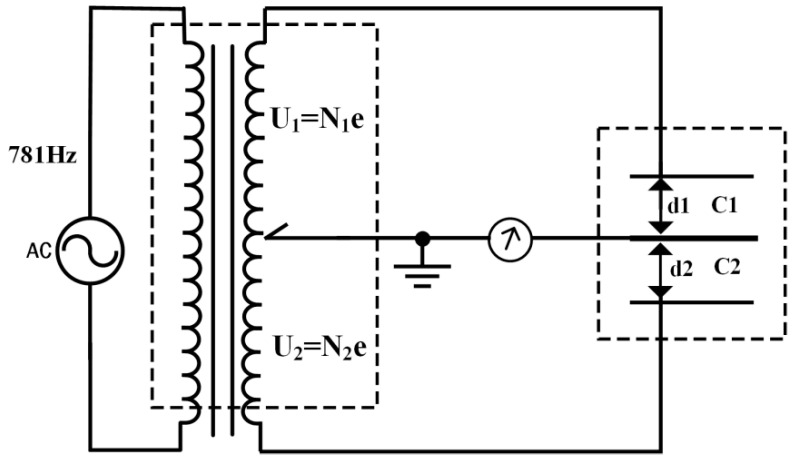
Schematic diagram of differential capacitance ratio bridge.

The differential capacitance sensor consists of three parallel metal pole plates with a fixed gap between the two sides of the pole plates, i.e., d1 + d2 = constant, and the center pole plate can be displaced with the deformation of the strain probe. The differential capacitance sensor utilizes a ratiometric bridge measurement circuit, the schematic diagram of which is shown in Figure 7 [25,26,27,28,29].

The ratio transformer in the figure is an inductively coupled ratio arm, wound by a high-permeability toroidal core. The inductively coupled ratio arm can be regarded as a 100,000-turn, per-turn tapping, and accurate voltage divider transformer with a voltage division accuracy of up to 10^−8^ or more and an output impedance of up to 10 mΩ or less. *N*_1_ and *N*_2_ represent the two parts of the ratio transformer with voltages corresponding to the number of turns, where *N*_1_ + *N*_2_ = N0 = 100,000 turns. The ratio transformer and the differential capacitance measurement bridge, when the bridge is in equilibrium and only considering the voltage amplitude, can be expressed as follows:

(9) $$\frac{C_{1}}{C_{2}}=\frac{U_{2}}{U_{1}}=\frac{N_{2}}{N_{1}}$$

Without considering the electric field edge effect, the capacitance value on both sides of the differential capacitor can be expressed as follows:

$$C_{12}=\frac{\varepsilon A}{3.6\pi d_{1}}$$

(10) $$C_{12}^{\prime }=\frac{\varepsilon A}{3.6\pi d_{2}}$$

where ε is the dielectric constant, A is the area of the pole plate, and $d_{1}$ and $d_{2}$ are the pole plate spacings. The capacitance expression is obtained by substituting it into Equation (6):

$$d_{1}=KN_{1}$$

(11) $$d_{2}=K(N_{0}-N_{1})$$

$$K=\frac{d_{1}+d_{2}}{N_{1}+N_{2}}$$

where K is the sensitivity factor of the measurement system, indicating the ratio of arm unit readings (turns) corresponding to the change in the displacement of the sensor center pole plate. The inductively coupled ratiometric arm comprises 100,000 turns, with the partial voltage being 1/N0 = 10^−5^. The effective spacing between the two pole plates on the outside of the differential capacitor is generally taken as 0.5 mm. Therefore, the displacement sensitivity coefficient is K = 5 × 10^−9^ m, which is only equivalent to 5 nanometers.

### 3.2. Differential Capacitive Micro-Displacement Sensor

**Figure 8:**
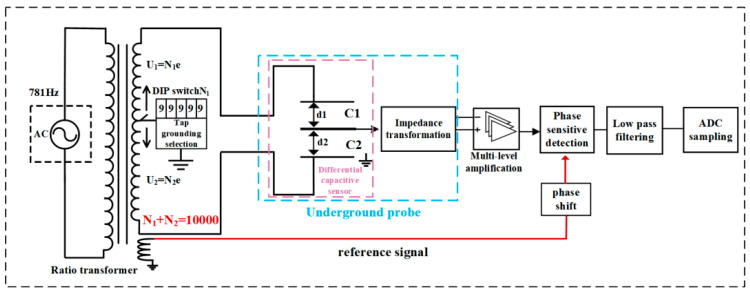
Ratio bridge measurement circuit for differential capacitance microdisplacement sensor.

As the ratio transformer adjusts the voltage according to the number of turns, with a minimum voltage division of 10^−5^ (equivalent to 100,000 turns for this system’s ratio transformer), the bridge achieves a minimum step of 10^−5^ for the voltage excitation. Thus, in actual regulation, the bridge generally does not reach an absolute balance. The actual measurement circuit is shown in Figure 8. The ratio transformer and differential capacitance sensors form an AC bridge, with the inductively coupled ratio arm using a 5-bit, 10-digit dipswitch-selective ground. This means that the dial plate reads the N1 value, at which point the middle pole plate voltage is grounded, resulting in an unbalanced signal output from the bridge.

Due to the high output impedance of the capacitor, the output signal of the middle pole plate first undergoes impedance transformation of the primary unit, and then, after signal amplification, phase-sensitive detection, and low-pass filtering, it is output to the AD data acquisition unit [30,31,32,33,34,35]. In the circuit, the amplitude of the AC excitation signal *U_AC_* is approximately 100 V. The signal undergoes amplification by the AC amplifier, phase-sensitive detection, and low-pass filtering, resulting in a total amplification of about 100 times. With the spacing of the capacitor pole plates being *d*_1_ + *d*_2_ = 0.5 mm, it can be deduced that $\frac{d_{1}+d_{2}}{{100U}_{AC}}=5\times 10^{-8}m/V$. A change of 1 V in the signal output indicates a displacement of 5 × 10^−8^ m of the capacitor plate. Thus, when the signal output voltage is 1 V, it indicates that the capacitor pole plate has been displaced by 5 × 10^−8^ m. The circuit depicted in Figure 8, through the phase-sensitive detector and low-pass filtering circuit, can achieve a stable output of 0.1 mV, enabling a displacement measurement resolution of 5 × 10^−11^ m.

On the other side of the bridge, the ratio arm tap ground point changes by one turn, corresponding to a one-bit change in the dial, resulting in a change of 0.1 V in the bridge imbalance signal. Therefore, the dip switch serves as a balancing and adjusting mechanism for the circuit, allowing the bridge imbalance signal to be adjusted to 0.05 V or less.

## 4. RZB-Type Three-Dimensional Drilling Strain Gauge Field Installation Observation

The RZB-type three-dimensional drilling strain gauge, installed and operated at the Gongxian seismic station in Sichuan Province, yielded improved observation results. The drilling depth at the Gongxian seismic station was 97 m, with drilling reaching 2 m below the complete granite layer. The total length of the strain probe was 3 m, and it was installed at a depth of 96 m. Special cement potting was utilized, with the cement potting extending to a depth of 82 m downhole, covering the probe by more than 10 m. The orientation of the horizontal strain sensors was as follows: the first element faced north at 153°, while the remaining second to fourth elements were arranged clockwise at 45° intervals. The first element of oblique strain was aligned with the first element of horizontal strain, angled diagonally upward at 45°. The second element of diagonal strain was aligned with the same direction as the third horizontal element, measuring upward at a 45° angle. The vertical strain measurement unit was positioned at the lowest part of the probe, measuring the vertical strain of the surrounding rock body.

**Figure 9:**
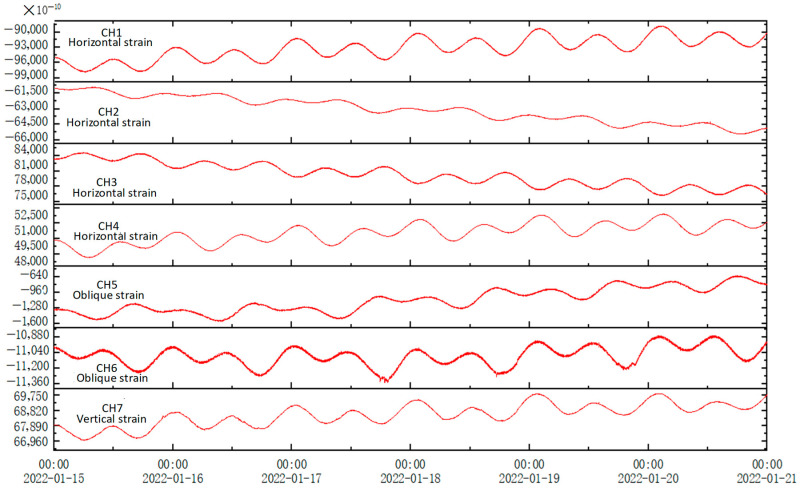
The strain measurement curve of a three-dimensional drill hole in Gongxian, Sichuan Province.

Due to the high sensitivity of borehole strain observation, the evaluation of the observation quality is generally based on the ability to observe clear, solid tidal and co-seismic responses [36]. The figure shows the three-dimensional borehole strain data curve observed at the Gongxian seismic station. As depicted in Figure 9, the three-dimensional borehole strain observation system at the Gongxian seismic station recorded clear solid tides and co-seismic responses across its four horizontal components, two oblique components, and vertical strain. The observed solid tide morphology exhibited a high signal-to-noise ratio that is consistent with the theoretical solid tide phase amplitude.

## 5. Venedikov Reconciliation Analysis of Strain Data [36,37,38,39,40,41]

To further quantitatively assess the data quality, the strain data underwent a solid tide reconciliation analysis. The Venedikov reconciliation analysis represents solid tide observations as a superposition of tidal waves.

(12) $$Y\left(t_{j}\right)=\sum_{i=1}^{n}H_{i}\cos\left[\omega_{i}t_{j}+\varphi_{i}\left(T_{j}\right)\right]+\phi (t_{j})$$

In Equation (12), $Y\left(t_{j}\right)$ represents the time observation sequence; $H_{i}$ denotes the observed amplitude of the tidal wave with angular frequency $\omega_{i}$; $\varphi_{i}$ represents the observed initial phase of the same tidal wave; $T_{j}$ represents the calendrical element at the central moment of the observation sequence; $t_{j}$ denotes the time interval from the central moment; and ϕ denotes the zero drift at $t_{j}$.

The strain data for each strain component at the Gongxian station from January 1 to 30 January 2022 were selected for a Venidikov reconciliation analysis. Using the odd and even digital filters designed by Venidikov (Equations (13) and (14)), the observed data series were digitally filtered to separate the diurnal waves from the semidiurnal waves. Subsequently, parameters such as the tidal factor and phase lag of each wave group were obtained using the principle of least squares.

Even digital filters:

(13) $$M_{j}^{\tau }=\sum_{i_{j}=-23.5}^{23.5}C_{t_{j}}^{\tau }y\left(t_{j}\right)$$

Odd digital filters:

(14) $$N_{j}^{\tau }=\sum_{i_{j}=-23.5}^{23.5}S_{t_{j}}^{\tau }y\left(t_{j}\right)$$

The solid tide curve observed by drilling strain actually superimposes the tides of surrounding waters. The tides of oceans, lakes, and rivers are not synchronized with the solid tides. The superposition of tides of waters on solid tides has a significant impact on the shapes of solid tides, especially the phase shapes. Therefore, in the evaluation of geophysical observation data, phase errors are only used as a reference. The tidal factor is the ratio of the tidal amplitude to the theoretical solid tide after filtering the observed data, and phase error is not involved in the calculation. According to the evaluation standard for solid tide measurement [36], the observed data and solid tide waves were compared with the M2 wave (semi-diurnal wave), with the largest amplitude being selected for comparison. Following the reconciliation analysis, the tidal factor’s mean squared error was calculated for evaluation. The error in the tidal factor of the M2 wave was within 0.05, meeting the measurement requirements.

The results of the M2 tidal wave reconciliation analysis for each strain component at the Gongxian station are presented in Table 1.

**Figure 10:**
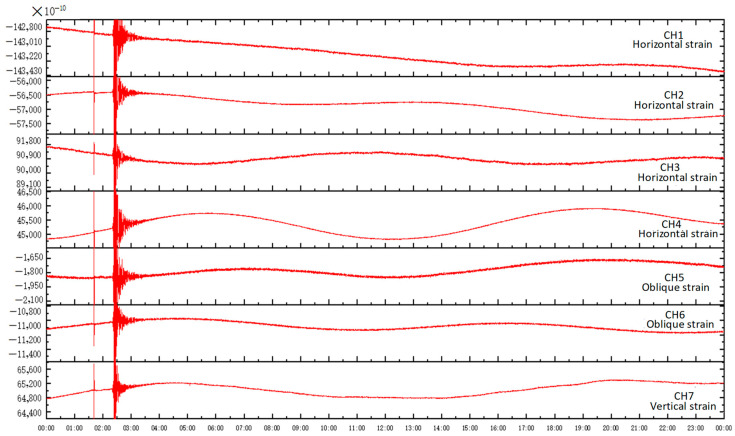
Co-seismic response recorded by the three-dimensional borehole strain observation system in Dove County.

The Gongxian Plateau is located along the Yangtze River with a well-developed water system around the station. About 400 m away from the station, there is also an artificial lake. Due to the influence of water tides, the phase deviation between the solid tide recorded in strain data and the theoretical solid tide is a bit large, with a maximum of 15°. However, the mean squared error of the tidal factor for M2 tidal waves is less than 0.05, which meets the observation standard. The observation resolution of the three-dimensional strain observation system reaches the order of 10^−10^ strains. Figure 8 shows the three-dimensional borehole strain recorded in Gongxian on 8 January 2022 at 1:45 p.m. during an earthquake with a magnitude of 6.9 in Menyuan County, Haibei Prefecture, Qinghai (37.77° N, 101.26° E) and at 2:09 p.m. during an earthquake with a magnitude of 5.1 in Menyuan County, Haibei Prefecture, Qinghai (37.78° N, 101.24° E). The observation data in Figure 10 show that each measurement item of the three-dimensional drilling strain observation system can record clear solid tides in the low-frequency band as well as strain seismic waves in the high-frequency band, with seismic phases clearly recorded.

## 6. Conclusions

Based on the RZB-type component (horizontal) drilling strain gauge, this article designs a vertical strain measurement unit with corrugated pipe structures and a diagonal strain measurement unit with telescopic guide rod structures, which are integrated with horizontal strain measurement probes to form the RZB three-dimensional drilling strain observation system.

1. The designed drilling vertical strain measurement unit adopts a corrugated pipe structure for the outer steel cylinder, with a vertical equivalent elastic modulus of about 10^3^ MPa, which is one order of magnitude lower than the coupled cement. The horizontal equivalent elastic modulus reaches more than 10^4^ MPa, which is one order of magnitude higher than the vertical direction. The horizontal deformation has a small impact on the vertical deformation and meets the requirements of vertical strain measurement.

2. The oblique strain probe adopts a linear strain measurement structure with a telescopic guide rod. The guide rod is connected to the outer cylinder of the probe through a soft rubber ring, achieving the observation of oblique linear strain.

3. The system adopts differential capacitive micro-displacement sensors and ratio bridge measurement circuits, with a strain measurement sensitivity of 10^−10^. The actual observation results at the Gongxian Seismic Station in Sichuan Province show that the horizontal, vertical, and oblique strain measurement units of the system can record clear solid tidal and co-seismic responses. The Venidikov reconciliation analysis shows that the recorded solid tide shape and amplitude are consistent with the theoretical solid tide and meet the requirements of geophysical field observation.

4. The theoretical formula for three-dimensional strain measurement has been derived. With the high sensitivity observation data of the RZB three-dimensional drilling strain observation system, three-dimensional strain problems can be solved, which is of great significance for regional crustal deformation, geodesy, crustal tectonic activity, and geophysical research [42,43,44].

## Figures and Tables

**Table 1:** Venidikov reconciliation analysis, M2 Wave, Dove County 3D Strain Observations, 1–31 November 2022.

Strain Channel	Tidal Factor	Tidal Factor Mean Squared Error	Tidal Phase Lag	Tidal Phase Lag Mean Squared Error
CH1 (Horizontal strain)	0.7005	0.0443	−9.5667	3.6243
CH2 (Horizontal strain)	0.8918	0.0346	−11.8036	8.5587
CH3 (Horizontal strain)	0.9821	0.0393	15.1021	9.5867
CH4 (Horizontal strain)	1.0082	0.0272	−2.4895	0.9458
CH5 (Oblique strain)	0.9165	0.0209	9.1653	5.6526
CH6 (Oblique strain)	0.8469	0.0449	−3.8329	3.6051
CH7 (Vertical strain)	1.1965	0.0388	8.1653	6.3626

## Data Availability

All data are contained within the article.
